# Editorial: Social technologies for inclusive development: multilevel policy and practices

**DOI:** 10.3389/frma.2025.1609399

**Published:** 2025-05-07

**Authors:** Kleinsy Bonilla, Efraín Bámaca-López, Susana Arrechea, Luis Guillermo Velásquez Perez

**Affiliations:** ^1^Organization for Women in Science for the Developing World, Trieste, Italy; ^2^School of Journalism, Faculty of Humanities, University of Santiago de Chile Santiago, Santiago, Chile; ^3^New Sun Road, Richmond, CA, United States; ^4^Área de Ciencia Política y de la Administración, Facultad de Derecho, Universidad de Salamanca, Salamanca, Spain

**Keywords:** social technologies, Latin America, inclusive development, science, technology and society (STS), grassroot knowledge, clean technology, technology & society, technology for development

This Research Topic presents a wide range of articles that position *social technology* as a key enabler of inclusive development across Latin America. By understanding social technology as situated knowledge and practice—rooted in local needs and shaped by social, cultural, and institutional contexts—this collection challenges traditional paradigms that treat technology as neutral, apolitical, or universally applicable. Instead, it embraces a Latin American perspective that highlights social technologies as tools for empowerment, cultural survival, and equity.

From case studies to policy analyses, the eight contributions gathered here reflect a transdisciplinary and practice-based approach. They explore how social technologies are used to address migration, food sovereignty, educational access, gender equity, and digital inclusion—often in regions with limited infrastructure and historical exclusion. These texts collectively illustrate how the design, implementation, and appropriation of technology can reflect or resist dominant power structures, and why inclusive development requires both digital innovation and social imagination.

Cabrera-Medina et al. analyze how digital technologies are deployed to manage migration across the United States, Mexico, Honduras, and Chile. While countries like the U.S. focus on biometric control systems, others such as Chile have adopted platforms like *Migrapp* to support migrant integration. The authors highlight the uneven landscape of migration technology and argue for a rights-based, people-centered approach—what they call “responsible design”—to ensure digital tools bridge gaps rather than reinforce exclusion.

In the agricultural domain, Apablaza critically examines how Industry 4.0 technologies, such as automation and artificial intelligence, are transforming Brazil's small-scale family farming. The analysis reveals that without public support, training, and inclusive governance, these tools risk exacerbating rural inequalities. Likewise, 10.3389/fcomm.2024.1505445 Lucki documents how limited infrastructure, cultural disconnection, and institutional fragility in Guatemala's western highlands undermine the potential of agricultural digitalization. Both contributions call for community-driven, context-sensitive strategies to avoid deepening the digital divide.

Grounded in food sovereignty and ancestral knowledge, Lugo Montilla and Águas present a compelling case from the Venezuelan Andes, where smallholder farmers use both traditional and modern techniques to rescue native potato seeds. The use of *tinopós* (underground storage spaces) and community breeding methods demonstrates how grassroots innovation can preserve agrobiodiversity, strengthen local economies, and challenge the technocentric logic of industrial agriculture.

Related to education, Bonilla et al. present INDESGUA as a successful community case of knowledge management as social technology. The non-profit connects rural youth and Indigenous communities in Guatemala with international scholarship opportunities, mediating between global funding organizations and local students. Through curated information, mentorship, and contextual guidance, INDESGUA overcomes structural barriers to higher education and fosters human capital development.

The use of digital tools for women's empowerment appears strongly in two articles. Figueroa and Alvarez Lemus evaluate a mentoring program for women in STEM in Mexico. The initiative combined online platforms, messaging groups, and virtual training, leading to measurable improvements in leadership, self-confidence, and professional development among participants. In parallel, Ortiz Osejo et al. present a mixed-methods study on Digital Community Centers (DCCs) in rural northern Guatemala. The study documents how internet access, digital skills training, and workshops on positive masculinities helped empower Indigenous Mayan women, expand their economic activities, and shift gender norms within the community. Despite persistent challenges like budget constraints and weak infrastructure, the DCCs illustrate the transformative potential of inclusive, community-led digital spaces.

The final contribution, Huete-Pérez et al., reflects on regional science, technology, and innovation (STI) policies in Nicaragua, Honduras, Guatemala, and El Salvador. The authors emphasize the chronic underinvestment in R&D and institutional weaknesses that hinder inclusive innovation. They call for systemic reforms, cross-sector collaboration, and the promotion of science diplomacy to build resilient innovation ecosystems that serve the region's development goals.

Taken together, these eight articles demonstrate that social technologies are not simply tools—they are processes shaped by values, power, and participation. Whether preserving native seeds, navigating migration, or mentoring young scientists, each contribution points to the importance of locally grounded, culturally relevant, and socially just approaches to technology adoption.

Across the board, several key themes emerge. First, many of these initiatives are driven by intermediary actors—platforms, organizations, or local networks—that translate between global resources and local needs. Second, capacity-building is central: communities must be empowered not just to access technology, but to adapt it, question it, and lead its use. Third, public institutions and policy frameworks matter. Without consistent investment, inclusive governance, and attention to equity, even the most promising technologies will fall short of their transformative potential.

This Research Topic reaffirms that inclusive development is not a byproduct of innovation—it must be a deliberate objective. Social technologies, when rooted in participation, culture, and context, offer powerful pathways to transform Latin America's most pressing challenges into opportunities for collective advancement.

We thank the authors, reviewers, and communities whose work enriches this collection. Their contributions not only inform academic discourse but inspire action among policymakers, practitioners, and local leaders seeking to co-create equitable, resilient, and digitally inclusive futures.

